# Use of propofol as an anesthetic and its efficacy on some hematological values of ornamental fish *Carassius auratus*

**DOI:** 10.1186/2193-1801-2-76

**Published:** 2013-03-04

**Authors:** Hosna GholipourKanani, Samaneh Ahadizadeh

**Affiliations:** Department of fisheries and natural resource, agriculture faculty, Gonbad Kavous University, Gonbad Kavous, Iran

**Keywords:** Anesthesia, Gold fish, Hematology, Ornamental, Propofol

## Abstract

The aim of this study was to determine the level of anesthesia attained in *Carassius auratus* using a propofol bath administration and using values of haematological profile of blood and examinations, to assess the effects of the fish exposure to that anaesthetic. Acute toxicity values of propofol for gold fish were found 96 h LC50 6.353 mg/L, 96 h LC1 2.966 mg/L and 96 h LC99 13.609 mg/L. Time to induce anesthesia in propofol experiment was significantly higher than Clove oil (p < 0.05), but there was no significant difference in recovery time between the experiments. No significant decrease was found in Total RBC, WBC, HCT, MCH, MCV and leukogram indices (p > 0.05). MCHC (%) level of propofol experiment (13.93 ± 1.36) showed significant (p < 0.05) decrease than Clove oil anesthesia (94.95 ± 24.50) and control (62.46 ± 21.90). Hb(g/dl) content (5.20 ± 0.73) showed decrease in propofol exposure compared with control (15.41 ± 4.76) and clove oil experiment (25.39 ± 5.73) (p < 0.05).

## Introduction

In recent years, different types of anesthetics are used to aid in the capture, handling, artificial reproduction, surgery procedures and transport of fish as an anti-stress in modern aquaculture ( Roubach et al. 2005 ).

A few number of anesthetics have proved effective in anaesthetisation of fish with its own advantages and drawbacks ( Velíšek et al. 2006 ). Till now, only MS-222 (tricaine methanesulfonate) is registered for use on food fish in the U.S. and the United Kingdom. However, aquaculture industry needs more compounds to be evaluated experimentally ( Coyle et al. 2004 ) and introduce on ornamental and food fish.

Some anaesthetics reduce or block the activation of the hypothalamic-pituitary-interrenal (HPI) axis associated with stressors and thus decrease or prevent the release of the stress hormone cortisol to the bloodstream of fish ( Hoskonen and Pirhonen 2006 ).

Anesthetics act on the central nervous system in such a way with placing the fish into an anaesthetic solution that is absorbed through the gills and enters the arterial blood, then with the returning of the anaesthetised fish to the fresh water, the anaesthetics or their metabolites are excreted via the gills ( Ross and Ross 1999 ).

Propofol (2,6 diisopropyl phenol) is an ultra-short–acting sedative agent with no analgesic properties, which provides sedative and anesthetic effects ( Kay and Stephenson 1980 ). Propofol is being widely used as an anaesthetic drug in human patients ( Andrews et al., 1997 ). It is reported to reduce both sympathetic and parasympathetic tone; however, it is not clear whether the changes in heart rate variability are associated with depth of anesthesia. It is generally considered safe for use in animals with renal or hepatic disease also most in instances of mild to moderate heart disease with appropriate monitoring and support.

Propofol is a short-acting, rapidly metabolized agent, which is characterized by a virtual lack of any cumulative effect and by rapid recovery after its administration in bolus doses or by continuous infusion. It provides a reliable, rapid and smooth induction of anaesthesia, adequate hypnosis and analgesia for surgical interventions and minimal suppression of vital organ functions. Moreover, recovery is observed to be rapid, uncomplicated and complete.

Propofol has been used for inducing anesthesia in reptiles such as green iguanas ( Knotkova et al. 2005 (Fleming et al. ) and some fish species such as *Acipencer oxyrinchus De soti*^2003^ ) and in spotted bamboo sharks () (Miller et al. *Chiloscyllium plagiosum*^2005^ ), but the efficacy and safety of any anaesthetic agent vary among species, life stages and environmental conditions, and more studies are needed to take advantage of this anesthetic in ornamental fish.

The aim of this study was to compare the effectiveness of propofol to that of the commonly employed clove oil, as an anesthetic for *Carassius auratus*. Gold fish were exposed to varying doses of propofol to determine the 96-h LC50, as well as exposure to both propofol and eugenol to observe differences in anesthesia onset and recovery times, to determine the proper dosage and to evaluate selected blood parameters during anaesthesia with propofol in gold fish (*Carassius auratus*).

## Method and material

In the study, propofol (pofol 1%) manufactured by the Dongkook Pharm Company (Choong cheong Book-Do, Korea) in 50 mL containers was used. Present research performed with the approval of an appropriate ethics committee INTL K3525.A35 B37 2000.

### Experimental fish

Approximately 207 sexually immature, gold fish were used, with an average weight of 8 ± 2 g (mean ± SD) and a mean fork length of 100 ± 20 mm. The fish population was distributed equally among ten 50–L holding tanks, each maintained at 22°C with well aeration. The fish were maintained on a lighting regimen representative of the local natural environment (13 L: 11 D) and fed twice daily to satiation with commercially available flaked tropical fish food. Tanks were siphoned once every second day and approximately 2 L of water was exchanged during each cleaning.

### Acute toxicity of propofol

Acute toxicity of propofol was ascertained by the OECD 203 “Fish, acute toxicity test” for the 96 h LC50 trials. At first Experimental fish (n = 72) were exposed to concentrations 0.5, 1, 2, 4, 8 and 16, mg/L dissolved in dechlorinated tap water and controls were placed in dechlorinated tap water with no tested substance added in five glass aquaria (50 cm × 26 cm × 30 cm) filled to a volume of 20 L. Twelve gold fish were randomly used for each concentration and for the control group in 2 replicate. The fish and its behavior, water temperature, pH and oxygen saturation were monitored throughout the tests at individual concentrations and in the control aquarium. The total mortalities, behaviors, temperature, and oxygen saturation were recorded every hour for the first 12 h of the experiment, every 3 h for the next 12 h, and every 6 h for the remaining 72 h. Fish were considered dead when there were no opercular beats observed for 15 continuously monitored min. This complete experimental protocol was replicated three times.

Mean lethal concentration at 96 h LC50 also 96 h LC1and 96 h LC99 was calculated from mortality rates over the period of 96 hours by the EPA probit analysis program version 1.5 software.

### Onset and recovery from anesthesia

The observations of stages 5-anesthesia onset were made using propofol and clove oil under the same experimental conditions. A 20- L experimental aquarium was maintained at a temperature of 22°C with oxygen saturation greater than 85%. Gold fish (n = 135) were randomly distributed into the experimental tank at the treatment concentrations of either 30 ppm clove oil ( Valisek et al. 2005 ) and 7 ppm propofol . Three replicates of 15 fish were used for each anesthetic concentration treatment of propofol, clove oil and control.

The times to achieve stage 5 of anesthesia were also recorded. Once an individual fish had reached the onset of stage 5 anesthesia, a dip net was used to immediately remove it from the tank. The fish was then transferred to a 20-L, well-oxygenated ‘recovery’ tank (i.e., no anesthesia present) maintained at 22°C and observed until it fully recovered. During this recovery period, the fish behavior was observed and times to recovery were recorded.

Once a fish had been used for a treatment, it was left in the recovery aquarium for approximately 1 day prior to being transferred back to a 50-L recovery holding tank for the remainder of a 14-day observational recovery period. Any abnormal behavior or mortalities were recorded during this 14-day recovery period. Anesthesia and recovery stages are presented in Table 1.

**Table 1:** Stages of anesthesia with clove oil in fish ( Keene et al., 1998 , modified from McFarland, 1959 ; Jolly et al. 1972 ) and Stages of recovery from anesthesia in fish ( Keene et al., 1998 modified from Hikasa et al., 1986 ) in fish

Stage	Behavior in anesthesia stages	Behavior in recovery stages
1	Normal Reacts to external stimuli; opercular rate and muscle tone normal	Decreased opercular movement
2	Light sedation Slight loss of reactivity to external visual and tactile stimuli; opercular rate slightly decreased; equilibrium normal	Partial recovery of equilibrium; partial recovery of swimming motion
3	Deep sedation Total loss of reactivity to external stimuli except very strong pressure;	Total recovery of equilibrium
4	slight decrease in opercular rate; equilibrium normal Partial loss Partial loss of muscle tone; increased opercular rate; reacts of equilibrium only to strong tactile and vibrational stimuli	Reappearance of avoidance swimming motion; reaction to external stimuli; behavioral response still stolid
5	Total loss Total loss of muscle tone and equilibrium; slow but regular of equilibrium opercular rate; loss of spinal reflexes	Swimming, rarely striking head firmly to sides or against bank of the tank
6	Loss of reflex Total loss of reactivity; opercular movements slow and reactivity irregular; heart rate very slow; loss of all reflexes	Total behavioral recovery; normal swimming

**Table 2:** Induction and recovery times for gold fish anesthetized with 7 ppm propofol and 30 ppm clove oil

Time (min)	propofol	Clove oil
Anesthesia (Stage 5)	7.40 ± .40*	4.26 ± .60
Recovery	8.52 ± .82	7.95 ± 1.21

Results are expressed as the mean ± SE; N = 36 for each anesthetic.

*p < 0.05.

### Haematological blood profile

For the haematological blood profile tests, in experimental I twenty of gold fish anesthetized with 7 ppm propofol were examined immediately after 10 min anaesthesia. In experiment II twenty of gold fish anesthetized with 30 ppm clove oil were examined immediately after 10 min anaesthesia and in Control group twenty of fish without any anesthesia were hematologicaly tested. Heparinized injection needles were used to take samples of blood from coudal vein of fish. To stabilize blood samples, aqueous solution of heparin sodium salt at 0.01 mL per mL of blood was used ( Svobodova et al. 1991 ).

The indices used to evaluate the haematological profile included the erythrocyte count (Er), haemoglobin concentration (Hb), haematocrit (PCV), mean corpuscular volume (MCV), mean corpuscular hemoglobin concentration (MCHC), erythrocyte haemoglobin (MCH), leukocyte count (Leuko) and the differential leukocyte count ( Svobodova et al. 1991 ).

Results of haematological examinations were tested by the variance analysis using the Statgraphics (ANOVA–Tukey Test) software.

## Results

### Acute toxicity of propofol

During the 96-hour LC50 tests, the mean water temperature was 22°C, pH was 7.5 and water oxygen levels were 75-85% saturation. Based on tests of acute toxicity to gold fish, the 96-hour lethal concentrations of propofol were determined (96 h LC50 6.353 mg/L, 96 h LC1 2.966 mg/L and 96 h LC99 13.609 mg/L). 96-h LC50 propofol of gold fish placed into the higher concentration (16 ppm) initially exhibited irritation, as evidenced by rapidly darting about the aquaria, the mortality of this concentration was 100% after 30 min. The first three concentrations (0.5. 1 and 2 ppm) survived the 96 h trial with 0% mortality. In concentration of 4 ppm deep sedation was observed after 10 min, with only one mortality after 24 h of exposure.

### Anesthesia and recovery

The time required to induce anesthesia using propofol (7 ppm) and clove oil (30 ppm) is shown in Table 2.

The time required to induce anesthesia using propofol was significantly higher than Clove oil (p < 0.05), but there was no significant difference in recovery time between the experiments. No mortality in anesthesia group was observed.

### Haematological parameters

Changes in the haematological parameters of gold fish in the control group and those exposed to propofol and clove oil are presented in Tables 3 and 4.

**Table 3:** **Effects of propofol and clove oil anaesthesia on haematological indices in gold fish**

Indices	Propofol	Clove oil	Control
Experiments			
Erythrocyte (×10^6^)	3.20 ± 0.63a	4.33 ± 0.30a	3.43 ± 0.43a
Hb (g/dL)	5.20 ± 0.73a	25.39 ± 5.73b	15.41 ± 4.76b
HCT (%)	31.80 ± 2.90a	27.40 ± 1.28a	26.60 ± 2.00a
MCV (fl)	137.4 ± 31.15a	64.98 ± 6.68a	83.42 ± 14.86a
MCH (pg)	19.25 ± 4.34a	59.60 ± 1.43a	47.84 ± 15.14a
MCHC (%)	13.93 ± 1.36a	94.95 ± 24.50b	62.46 ± 21.90b
Leuko ( ×10^4^)	5.60 ± 0.97a	2.37 ± 0.95a	2.97 ± 0.63a

Groups with different alphabetic superscripts differ significantly at *p < 0.05* (ANOVA).

Results are expressed as the mean ± SE; N = 20 for each concentration of anesthetic.

**Table 4:** **Effects of propofol and clove oil anaesthesia on differential leukocyte counts in gold fish**

Indices (%)	Propofol	Clove oil	Control
Experiments			
Lymphocytes	92.80 ± 0.66a	92.00 ± 0.54a	93.80 ± 0.37a
Monocytes	0.20 ± 0.20a	0.00 ± 0.00a	0.00 ± 0.00a
Neutrophil	6.60 ± 0.60a	7.80 ± 0.37a	6.00 ± 0.44a
Eosinophil	0.40 ± 0.24a	0.20 ± 0.20a	0.20 ± 0.20a

Groups with different alphabetic superscripts differ significantly at *p < 0.05* (ANOVA).

Results are expressed as the mean ± SE; N = 20 for each concentration of anesthetic.

No significant decrease was found in Total RBC, HCT, WBC, MCH, MCV and leukogram indices (p > 0.05). MCHC level of propofol experiment (13.93 ± 1.36) showed significant (p < 0.05) decrease than Clove oil experiment (94.95 ± 24.50) and control (62.46 ± 21.90). Decrease in Hb content (5.20 ± 0.73) was observed in propofol exposure compared with control (15.41 ± 4.76) and clove oil experiment (25.39 ± 5.73) (p < 0.05).

## Discussion

Anaesthetics are necessary for many procedures in aquaculture. The analysis of blood parameters is one of the most valuable methods of anaesthetics evaluation, because it has been shown that the physiological effects of anaesthetics are species-specific and age-dependent ( Anver Celik 2004 ). Because species may differ widely in their response to anaesthetics, screening of their use is necessary.

In this study acute toxicity of propofol to *Carassius auratus* is investigated from the point of view of propofol use as an anaesthetic, with anaesthetizing baths.

Hematological parameters can provide needed information on the physiological status of fishes, and help the aquaculture and research personnel to make proper decisions to increase the survival of fishes

Propofol (Diprivan®, Rapinovet®, Propoflo®), an alkyl phenol hypnotic has been investigated as a widely used intravenous anaesthetic in veterinary practice. Use of propofol as a sole anaesthetic produced effective general anaesthesia in different domestic animals ( Duke et al., 1997 ; Lin et al., 1997 ; Carroll et al., 1998 ; Bayan et al., 2002 ; Zama et al., 2003 ; 2005 ) Propofol used as an anaesthetic agent in lizards showed a rapid onset of action. Following intravenous administration in green iguanas, the onset of anaesthesia maybe expected within several minutes ( Bennett et al. 1998 ).

Guénette et al. ( 2008 ) determined the level of anesthesia attained in *Xenopus laevis* frogs using a propofol bath administration. An appropriate anesthetic dose was determined to be 88 mg/L for 15 min.

There are a few experimental papers reporting the effect of propofol on the fish species. According to FDA guidelines acute toxicity to rainbow trout (*Onchorhynchus mykiss*) and bluegill sunfish (*Lepomis macrochirus*) (FDA guideline 4.11, flow-through –no aeration) is 96 h LC50 = 0.37 mg/L and 96 h LC50 = 0.62 mg/L respectively. The bio concentration factor (BCF) has been determined for carp, *Cyprinus carpio*, and the results reported as: (BCF) 28 day = 27 (at 2 μg/L) and (BCF) 28 day = 26 (at 0.2 μg/L).

The bio degradability of propofol has been assessed according to the OECD guideline 301 F, the results showed >91% removal of propofol from the aqueous phase.

Fleming et al. ( 2003 ) evaluated propofol for short–term immobilization of Gulf mexico sturgeon (*Acipencer* Oxyrinchus de soti) and it was observed that the group receiving intra venous propofol (6.5 mg/kg body weight, i.v.) was in a light plane of anesthesia within 5 min after drug administration.

The effect of propofol on haematological parameters is reported in sheep ( Brzeski et al., 1994 ), dogs ( Gill et al. 1996 ), ewes ( Handel et al. 1991 ), rabbits ( Mazaheri-Khameneh et al. 2012 ) and horses ( Mama et al., 1998 ), no other data on the blood profiles in the fish species anaesthetized with propofol are available in the literature.

Gold fish exposed to propofol showed lower Hb and MCHC. It is known that propofol induces moderate systemic hypotension, arterial vasodilatation and venodilatation ( Branson and Gross, 1994 ).The lower concentration of Hb, could be explained by haemodynamic changes and re-distribution of blood cellular elements in the vascular bed.

In conclusion, the result of this study indicated that propofol (7 ppm) can induce safe and valid anaesthesia in gold fish. However, it seems that further studies on different dosage, also measuring more haematological and biochemical parameters in the gold fish and other (non-food) ornamental fish following anaesthesia with propofol are needed.
